# The Sedentary–Stress Paradox in Esports: Neuroendocrine, Metabolic, and Endothelial Pathways to Cardiovascular Risk

**DOI:** 10.3390/ijms27209365

**Published:** 2026-10-21

**Authors:** Amelia Tero-Vescan, Ruxandra Ștefănescu, Bianca Eugenia Ősz, Mădălina Buț, Amalia Pușcaș, Ylenia Pastorello, Mark Slevin

**Affiliations:** 1 Biochemistry Department, George Emil Palade University of Medicine, Pharmacy, Science, and Technology of Târgu Mureș, 38th Gh. Marinescu Street, 540139 Târgu Mureş, Romania; amelia.tero-vescan@umfst.ro (A.T.-V.);; 2 Pharmacognosy and Phytotherapy Department, George Emil Palade University of Medicine, Pharmacy, Science, and Technology of Târgu Mureș, 38th Gh. Marinescu Street, 540139 Târgu Mureş, Romania; 3 Pharmacology and Clinical Pharmacy Department, George Emil Palade University of Medicine, Pharmacy, Science, and Technology of Târgu Mureș, 38th Gh. Marinescu Street, 540139 Târgu Mureş, Romania; 4 Department of Anatomy and Embryology, George Emil Palade University of Medicine, Pharmacy, Science, and Technology of Târgu Mureş, 540142 Târgu Mureş, Romania; ylenia.pastorello@umfst.ro; 5 Center for Advanced Medical and Pharmaceutical Research, George Emil Palade University of Medicine, Pharmacy, Science, and Technology of Târgu Mureș, 38th Gh. Marinescu Street, 540139 Târgu Mureş, Romania; mark.slevin@umfst.ro

**Keywords:** esports, gaming, sedentary behavior, neuroendocrine stress, HPA axis, sympathetic nervous system, sarcopenic obesity, endothelial dysfunction, nitric oxide, cardiovascular risk

## Abstract

Esports and gaming resemble traditional sports and physical activity because both involve sustained cognitive and emotional stress; however, this stress occurs against a background of prolonged sedentary behavior. This narrative review examines the neuroendocrine, metabolic, and endothelial consequences of this “stress without movement” phenotype and its potential implications for long-term cardiovascular health. Activation of the hypothalamic–pituitary–adrenal (HPA) axis and sympathetic nervous system disrupts sleep and circadian rhythms. Continuous neuroendocrine activation may impair muscle–adipose tissue crosstalk and nitric oxide-dependent endothelial function. Although direct longitudinal evidence linking gaming to cardiovascular disease is currently lacking, these convergent mechanisms identify prolonged sedentary gaming as a potential cardiovascular risk amplifier, particularly when combined with occupational sitting, sleep disruption, adiposity, and other cardiometabolic risk factors.

## 1. Introduction

Gaming is no longer perceived as a mere recreational activity. Gaming has evolved into a global phenomenon including both individual and team-based gaming, with players often engaging in prolonged sessions of 6–12 h per day, either independently or within organized teams and guilds employing complex, coordinated strategies. These structures closely resemble traditional sports, where selection is based on performance metrics, skill level, and competitive ability.

Esports (electronic sports) refers to organized competitive video gaming in which individuals or teams compete against one another, often involving professional training, established rules, and performance-based rankings. Unlike recreational gaming, esports emphasizes competitive performance, strategic decision-making, and coordinated cognitive–motor skills. The global esports industry has been estimated at approximately USD 25 billion, highlighting its rapid expansion and increasing social impact [1]. The elite esports athlete phenotype combines high cognitive load (prolonged attention and rapid decision-making), sustained emotional stress (performance under pressure and precise motor coordination), and minimal physical activity, representing an extreme form of sedentary behavior [2]. This sedentary profile parallels prolonged desk-based work, where extended periods of sitting have experimentally been associated with reduced lower-limb blood flow and shear stress, impaired vascular function, and adverse postprandial metabolic responses, providing a well-established physiological framework for understanding the potential health consequences of similarly prolonged gaming sessions [3].

These combined factors describe a physiological and biochemical paradox: intense neuroendocrine activation of the hypothalamic–pituitary–adrenal (HPA) axis and sympathetic nervous system occurring in the absence of significant muscular activity [4]. At the molecular level, this disruption leads to low-grade chronic inflammation, altered adipokine profiles (leptin resistance and reduced adiponectin), mitochondrial dysfunction, and oxidative stress, forming the core mechanism behind metabolic syndrome and its associated comorbidities [5].

Sedentary behavior is associated with excessive screen time, described in this review as “stress without movement”, and it may contribute to metabolic disorders. This is reflected in reduced values of exercise cardiac power (ECP), defined as the ratio between directly measured maximal oxygen uptake (VO_2_max) and peak systolic blood pressure during gaming sessions, thereby increasing cardiovascular risk, as demonstrated by a population-based cohort study [6]. Although these findings cannot be directly extrapolated to esports participants, they provide a physiological basis supporting the plausibility of an association between gaming-related stress and cardiovascular vulnerability. Prolonged sedentary behavior and insufficient physical activity may contribute to unfavorable changes in body composition, including reduced relative muscle mass and increased adiposity, potentially increasing susceptibility to a sarcopenic obesity phenotype. A population-based NHANES 2011–2018 cohort, including 5412 children and adolescents indicated an overall 15.6% prevalence of sarcopenic obesity [7], while the German Children’s Health Interventional Trial (CHILT III) showed, in a cohort of overweight and obese children and adolescents, that approximately 70% fulfilled the criteria for sarcopenia. The data suggested that unfavorable alterations in body composition may develop early in life and could be exacerbated by prolonged sedentary behaviors such as esports participation [8].

The aim of this narrative review is to integrate evidence on recurrent neuroendocrine stress during gaming, characterized by activation of the HPA axis and sympathetic nervous system and its consequences for sleep quality and duration, with evidence on endothelial dysfunction and lack of physical activity. We propose that this combination of neuroendocrine stress and prolonged sedentary behavior, conceptualized as “stress without movement,” may create a phenotype theoretically predisposed to an increased risk of cardiovascular disease and sarcopenic obesity.

## 2. Literature Search Strategy

This narrative review was developed through a literature search focused on the neuroendocrine, metabolic, and cardiovascular consequences of esports participation and prolonged sedentary gaming. Relevant scientific publications were identified through searches of PubMed/MEDLINE, Scopus, and Web of Science, using combinations of the following keywords: “esports”, “electronic sports”, “video gaming”, “sedentary behavior”, “cognitive stress”, “hypothalamic–pituitary–adrenal axis”, “cortisol”, “sympathetic nervous system”, “sleep disruption”, “circadian rhythm”, “sarcopenic obesity”, “myokines”, “adipokines”, “endothelial dysfunction”, “nitric oxide”, and “cardiovascular risk”.

Priority was given to original experimental and observational studies investigating physiological responses to competitive or recreational gaming, including changes in cortisol secretion, heart rate variability, blood pressure, sleep characteristics, body composition, and energy expenditure. Given the limited availability of direct mechanistic investigations in esports athletes, complementary experimental studies involving prolonged sedentary behavior, psychological stress, glucocorticoid exposure, skeletal muscle inactivity, and endothelial function were also considered to provide a mechanistic framework.

Studies were selected based on their relevance to the proposed interactions between neuroendocrine activation, metabolic dysregulation, muscle–adipose tissue communication, and vascular function. Attention was given to distinguishing findings directly demonstrated in esports participants from mechanisms extrapolated from other human or experimental models. The evidence was synthesized qualitatively to identify convergent molecular pathways and formulate the proposed “stress without movement” framework. No quantitative meta-analysis or formal assessment of study quality was performed, consistent with the narrative scope of this review.

To clarify the hierarchy of evidence, the findings discussed in this review were distinguished according to their experimental context: (i) direct evidence from esports athletes or recreational gamers, including observational and acute experimental studies; (ii) indirect human evidence from studies investigating prolonged sitting, physical inactivity, psychological stress, or sleep restriction in non-gaming populations; and (iii) mechanistic evidence from animal models, cell cultures, and experimental interventions involving immobilization or glucocorticoid administration. While direct gaming studies provide population-specific physiological observations, indirect human and preclinical studies primarily support the biological plausibility of the proposed pathways. Importantly, mechanistic findings from these models should not be interpreted as direct evidence of chronic metabolic or cardiovascular dysfunction in esports participants. Table 1 summarizes the available evidence according to its source and relevance to esports, highlighting the limitations of translating experimental findings into long-term cardiometabolic outcomes.

## 3. Esports as a Model of Chronic Cognitive–Sedentary Stress

### 3.1. Behavioral and Physiological Characteristics

Both traditional sports and esports are characterized by periods of intense stress, strategic decision-making, and complex psychomotor coordination. However, the fundamental differences are the absence of substantial motor activity, which classifies esports as a predominantly sedentary behavior and the temporal pattern of exposure: whereas competitive events in traditional sports are generally time-limited and interspersed with periods of recovery, esports players may engage in prolonged training and competitive gaming for 6–10 h per day.

Since 2023, Bubna et al. have emphasized the need to differentiate between recreational players, called “amateur players” in this review, and participants in esports competitions, proposing the term “esports athlete” for people who participate in competitive video games with the aim of obtaining an official rank within the game or who compete in organized competitions [31].

Studies report a paradoxical phenomenon: there is a clear difference in terms of biological parameters between esports athletes and non-players, but this difference also exists between esports athletes and amateur players. Compared with age-matched controls (mean age approximately 20 years), esports athletes competing in the American Collegiate East Coast Esports League exhibited lower number of steps taken per day and differences in body composition, particularly higher proportion of body fat relative to lean mass [21].

Many players, whether professional gamers affiliated with top-tier esports organizations or amateur gamers, report musculoskeletal complaints, particularly involving the shoulders, neck, hands, and wrists, as well as at least one ocular symptom. Although no significant differences have been reported in body mass index (BMI), body fat percentage, or waist circumference, the prevalence of grade I or II obesity and a high visceral fat percentage was greater among amateur players [32].

Professional and semi-professional esports athletes, as well as amateur players, often dedicate a considerable number of hours per day to gaming activities, experiencing chronic cognitive–sedentary stress that represents an intermediate physiological context between traditional athletic competition and passive sedentary behavior. Among 1038 predominantly male esports athletes and amateur players (mean age, 23.0 ± 5.4 years), most participants reported an average gaming time of approximately 230 min per day, in addition to time spent watching gaming-related livestreams and videos, a pattern that became even more pronounced during the COVID-19 pandemic [2].

An additional aspect of videogaming is its rewarding and pleasurable nature, which may coexist with the cognitive and neuroendocrine stress associated with competitive performance. Experimental evidence indicates that videogaming engages dopaminergic reward pathways, particularly within the striatum. Positron emission tomography (PET) with [^11^C]raclopride demonstrated changes consistent with endogenous dopamine release in the human striatum during videogame performance [33]. Reward processing in 80 individuals classified as casual gamers, regular gamers, or individuals with internet gaming disorder was investigated using electroencephalography during a monetary incentive delay task. Individuals with gaming disorder exhibited increased cue-related P300 amplitudes and reduced stimulus-preceding negativity compared with casual gamers, probably because of heightened attentional sensitivity to reward cues but altered cognitive anticipation of reward outcomes. Importantly, gaming experience, rather than addiction symptom severity, was associated with increased neural responsiveness to reward-predicting cues [34].

These dopaminergic processes may contribute to motivation, enjoyment, and sustained gaming engagement. Endogenous opioid signaling, including β-endorphin-related pathways, may also contribute to pleasurable experiences; however, direct experimental evidence demonstrating endorphin release during esports participation remains limited. Therefore, gaming-related reward activation and neuroendocrine stress should not be considered mutually exclusive, although their combined effects on long-term cardiovascular health remain insufficiently investigated.

Throughout this review, the distinction between esports athletes and amateur players is maintained according to the characteristics of the populations investigated in the cited studies. Findings obtained from recreational gamers are not assumed to be directly generalizable to competitive esports athletes, particularly given potential differences in training schedules, competitive exposure, physical activity, and lifestyle characteristics. Where studies include mixed or insufficiently characterized gaming populations, this limitation is explicitly acknowledged.

### 3.2. Sleep Disruption and Circadian Misalignment

Sleep is a fundamental biological process involved in neuroendocrine recovery, metabolic homeostasis, immune regulation, and cognitive performance. A crossover study, conducted on 12 healthy, nonobese adults (9 males, ages 19–39 years), exposed to either sleep restriction (4 h/night) or normal sleep (9 h/night), for 14 days showed that participants experienced increases in weight, total abdominal fat, both subcutaneous and visceral in case of sleep deprivation [16].

Prolonged exposure to blue light, psychological arousal, and irregular schedules produces circadian rhythm imbalance, with HPA activation and important metabolic and cardiovascular consequences. The sleep profile of 17 professional esports first-person shooter (FPS) players (20 ± 3.5 years) was monitored for 7–14 days using continuous wrist activity monitors and subsequently assessed using sleep questionnaires. A markedly delayed circadian phenotype was identified, characterized by a median bedtime of 03:43 and wake time of 11:24, a total sleep duration of only 6.8 h/night, a sleep latency of 20.4 min, and wake after sleep onset (WASO) of 47.9 min. Depressive symptoms were significantly correlated with sleep fragmentation and daily training duration, which reached 13.4 h/day in some players [17].

Following a nightly gaming session involving FPS, role-playing game (RPG), or real-time strategy games for at least 200 min/day, compared with an evening without electronic device use, sleep duration and quality, monitored using a wrist activity monitor and assessed with the Pittsburgh sleep quality index (PSQI), decreased by approximately 39 min, while stress, assessed through heart rate variability (HRV), increased significantly among 44 amateur esports players (25.2 ± 4.6 years) [18].

In addition to changes in sleep duration, e-sports athletes frequently exhibit alterations in circadian rhythm. Prolonged exposure to blue light emitted by screens in the evening suppresses pineal melatonin secretion by activating intrinsically photosensitive retinal ganglion cells and subsequent signaling to the suprachiasmatic nucleus (SCN). Reduced melatonin availability delays sleep onset and shifts circadian rhythms toward a delayed phase and contributes to chronic circadian misalignment. Experimental and clinical evidence indicates that nocturnal exposure to blue light impairs both sleep quality and sleep duration, while negatively affecting cognitive function, mood, and metabolic regulation (Figure 1) [19].

Although findings regarding differences in overall sleep quality between esports athletes and amateur players remain inconsistent, significant differences have been reported in total sleep duration, potentially reflecting the academic or occupational obligations of amateur players. A study of 90 Japanese gamers (22.4 ± 6.6 years), including 35 esports athletes, found no significant differences in overall sleep quality, but esports athletes exhibited a significantly delayed sleep pattern, characterized by later bedtimes and wake times, whereas amateur gamers tended to have a shorter total sleep duration. Moreover, gaming between 03:00 and 08:59 was independently associated with a more than twofold increased risk of poor sleep quality, as assessed by the PSQI [20].

Given these data, it can be concluded that sleep disturbance and circadian rhythm changes as a direct consequence of gaming could be considered the link between chronic cognitive stress, neuroendocrine activation, metabolic dysfunction, and cardiovascular vulnerability.

## 4. Neuroendocrine Mechanisms Linking Gaming-Related Stress to Metabolic Dysregulation

### 4.1. Cortisol Dynamics and Glucocorticoid Receptor Desensitization

Esports and gaming, whether played individually or in teams, for performance or recreation, involve the presence of stress factors specifically anticipatory pressure, cognitive-motor coordination, performance expectations, and rapid decision-making that activate the HPA axis, the main endocrine-metabolic translation mechanism. For example, salivary cortisol levels increased significantly at the end of a gaming competition in both sexes, being more pronounced in the case of winners [9].

Interestingly, Zilioli and Watson showed the same discrepancy in salivary cortisol levels for 70 male players aged (19.9 ± 2.27 years) in a manipulated Tetris game in which winner and loser conditions were randomly assigned. Cortisol values showed not only changes during the competition but also significant differences in the case of losers in whom the decline in salivary values in post-competition was higher compared to the winners. This was likely caused by significant interaction between the hypothalamic–pituitary–gonadal (HPG) and HPA axes and the competition effect on testosterone [10].

Anticipatory psychophysiological activation preceding competition is highly important in professional esports players as pre-game cortisol levels and cognitive anxiety scores were significantly higher compared to non-gamer controls, while post-game measurements showed no significant differences between groups in a multiplayer online battle arena (MOBA) competition [11].

From a biochemical perspective, cortisol secretion increases after approximately 10–15 min of continuous stress, values are more clearly increased after 20–30 min and can reach a peak at 30–60 min because even though corticotropin-releasing hormone (CRH) and adrenocorticotropic hormone (ACTH) secretion is rapid, cortisol production at CSR level is slow and has the role of keeping the body “on alert” (Table 2) [35,36]. Therefore, a very important factor to consider when talking about HPA axis activation is total game time in a session. A gaming session of 30 min did not significantly influence oxygen uptake, energy expenditure, stroke volume, lactate, and carbon dioxide output even though pre- vs. post-game cortisol levels were significantly higher in case of amateur players aged 23.1 ± 3.0 years who regularly played 12.3 h/week [12]. The acute cortisol responses observed during competitive gaming reflect transient HPA-axis activation and should not be interpreted as evidence of chronic hypercortisolism, glucocorticoid receptor resistance, or persistent HPA-axis dysregulation in esports participants.

Glucocorticoids act primarily through the intracellular glucocorticoid (GR) and mineralocorticoid (MR) receptors (Figure 2). In the absence of ligand, these receptors are associated with molecular chaperones and co-chaperones, including HSP90, FKBP5, and BAG1, which regulate receptor stability, ligand binding, and intracellular trafficking [37]. Upon glucocorticoid binding, activated receptor complexes translocate to the nucleus, where they interact with glucocorticoid response elements (GREs) and transcriptional coregulators to activate or repress target gene expression [38]. GR and MR signaling can also modulate transcription through interactions with other transcription factors, including cAMP response element-binding protein (CREB) and nuclear factor kappa B (NF-κB), thereby influencing genes involved in neuroplasticity, such as brain-derived neurotrophic factor (BDNF), as well as inflammatory pathways [39]. Beyond these genomic effects, glucocorticoid signaling influences mitochondrial function, oxidative phosphorylation, Ca^2+^ homeostasis, autophagy, and apoptosis [40]. At the synaptic level, glucocorticoids further modulate GABAergic and glutamatergic neurotransmission, including N-methyl-D-aspartate receptor (NMDAR)-dependent signaling and intracellular Ca^2+^ dynamics. Persistent dysregulation of these interconnected pathways may contribute to impaired mitophagy and proteostasis, oxidative stress, neuroinflammation, and neuronal dysfunction [41].

Another important aspect to consider is the phenomenon of receptor desensitization, so-called glucocorticoid receptor resistance (GRR) that occurs through negative feedback on the HPA axis at chronically elevated serum cortisol levels. Even though there are no studies showing the effect of chronic stress during videogaming on GRR, it is generally accepted that sensitivity to corticosteroids presents significant differences between individuals. For example, healthy adults of both sexes exposed to a rhinovirus (nasal drops with RV39) presented different levels of IL-1β, IL-6, and TNF-α in nasal secretions, with those with GRR developing more inflammation. Therefore, paradoxically, although glucocorticoids are used in medical practice as potent anti-inflammatory and immunosuppressants, chronic or repeated glucocorticoid exposure may paradoxically promote inflammation through impaired glucocorticoid receptor signaling [42].

### 4.2. Chronic Glucocorticoid Exposure and Stress-Induced Metabolic Reprogramming

Acute secretion of glucocorticoids is a normal, adaptive response to stress that provides the body with adequate energy substrate. Chronically secreted, glucocorticoids subject the body to a continuous low-level metabolic stress and promote the development of a specific phenotype characterized by hyperglycemia, insulin resistance, dyslipidemia with visceral fat deposition, and skeletal muscle protein catabolism, providing amino acid substrates for hepatic gluconeogenesis.

One of the most important effects of chronic glucocorticoid exposure is the promotion of gluconeogenesis with a decrease in its peripheral utilization. Through its canonical genomic mechanism of action, cortisol promotes transcription of phosphoenolpyruvate carboxykinase (PEPCK) and glucose-6-phosphatase, key enzymes for glucose metabolism, with concomitant release of glucose from the liver into the blood, determining insulin secretion from beta-pancreatic cells [13].

In addition, glucocorticoids inhibit insulin receptor substrate-1 (IRS-1)/phosphoinositide 3-kinase (PI3K)/protein kinase B (Akt) signaling and glucose transporter type 4 (GLUT4) vesicle exocytosis toward the cell membrane, decreasing glucose uptake in peripheral cells, which is one of the mechanisms for insulin resistance. Studies performed on cultured adipocytes showed that acute use of dexamethasone (2 h) reduced basal glucose transport by 65% mainly by preventing GLUT4 translocation to the membrane, while prolonged exposure (24 h) reduced peripheral glucose utilization by 77%, also inhibiting insulin-stimulated transport by 55% [25].

The influence of glucocorticoids on AMP-activated protein kinase (AMPK) is tissue-dependent, thus in the skeletal muscles, they decrease AMPK activity and mitochondrial oxidative capacity, while in the macrophages they activate AMPK to successfully resolve inflammation and induce tissue-repair. For example, male Sprague-Dawley rats showed significantly impaired skeletal muscle AMPK signaling following 2 weeks of treatment with corticosterone. Tissue also showed reduced capacity for electrical stimulation of α2-AMPK, with a concomitant decrease in phosphorylation of the glucose-regulatory protein TBC1D1, increased intramuscular malonyl-CoA accumulation, (indicating impaired fatty acid oxidation), and reduced insulin sensitivity [26].

In the skeletal muscles, chronic exposure to glucocorticoids is responsible for steroid-induced myopathy, through targeted action on fast-twitch (Type II) fibers where it inhibits insulin and insulin-like growth factor I (IGF-I)-induced protein synthesis and promotes their degradation, as was demonstrated in patients with Cushing’s syndrome. Adult (6–8 months) and old (18 months) male Sprague–Dawley rats treated with dexamethasone for 5 days in old rats and 6 days in adults to generate a similar degree of effects, presented reduced insulin and IGF-I anabolic effects on muscle protein synthesis, an effect much more evident in old rats [27].

In the adipose tissue, glucocorticoids exhibit paradoxical effects, from promoting lipolysis under physiological conditions, mechanisms explained by canonical effects on the GR, to pathological lipid accumulation in the abdomen. A study that followed the acute (1–4 h) exposure to high levels of glucocorticoids of 3T3-L1 white adipocytes paradoxically showed the suppression of lipolysis in WAT by decreasing hormone-sensitive lipase (HSL) activity, through a non-canonical post-translational mechanism independent of classical GR signaling as was highlighted by the use of a GR inhibitor [28].

Chronic hyperadrenocorticism is responsible for the activation of 11β-hydroxysteroid dehydrogenase type 1 (11β-HSD1), the enzyme that converts cortisone into active cortisol in the tissues, independently of circulating cortisol concentrations. This transformation is responsible for metabolic stress, especially in the liver and in adipose tissue, with lipid accumulation as demonstrated in a small cohort of 8 dogs with pituitary-dependent hyperadrenocorticism which presented significantly increased values of this enzyme in visceral adipocytes, while the increased activity of this enzyme in the brain produces memory impairment and post-traumatic stress disorder (PTSD)-like memories [43].

All these data suggest that chronic exposure to glucocorticoids under stressful conditions may promote a metabolic phenotype characterized by impaired glucose regulation, reduced skeletal muscle oxidative capacity, and altered lipid metabolism. These effects could potentially be exacerbated by prolonged sedentary behavior associated with esports and videogaming. However, direct experimental evidence demonstrating these metabolic alterations in esports athletes is currently lacking, and their relevance to this population remains hypothetical.

## 5. Activation of the Sympathetic Nervous System and the Effects of Catecholamines in Sedentary Conditions

Another important aspect in gaming is the acute activation of the sympathetic nervous system, with secretion of catecholamines from the adrenal medulla, a rapid flight or fight response, with increased alertness and facilitation of cognitive performance.

Under conditions of acute or chronic stress, increased release of catecholamines (adrenaline and noradrenaline) activates cardiac β_1_-adrenergic receptors, causing tachycardia, increased myocardial contractility and cardiac output. Prolonged sympathetic stimulation can favor the occurrence of pathologies such as hypertension, cardiac remodeling, endothelial dysfunction and increased risk of cardiovascular disease [44].

Bennett et al. investigated the cardiovascular autonomic response in 19 men (18–30 years) who participated in competitive MOBA sessions lasting 1.5–2.5 h. Participants wore Hexoskin Smart Shirts^®^, which continuously recorded RR and NN intervals to assess HRV, using the standard deviation of RR intervals (SDRR) as the main parameter. The results showed considerable individual variability in HRV throughout the game, suggesting important differences in stress response between players. Winning teams showed lower mean SDRR values compared to losing teams, indicating sympathetic activation and a more pronounced physiological stress response [45].

Exergaming (exercise gaming) refers to physically interactive video games that require bodily movements or muscular contractions to control gameplay, combining physical activity with interactive gaming. Unlike conventional sedentary gaming, exergaming increases energy expenditure and may induce physiological responses comparable to those associated with exercise, depending on game intensity and modality. A study by Bonetti et al. evaluated the acute physiological responses to isometric resistance-based exergaming compared to conventional gaming in 32 healthy men aged 18–30 years, randomly assigned to a group that used a conventional controller and an experimental group that used a whole-body isometric contraction-controlled device (Exerstation) during competitive action/arcade gaming sessions. Compared to conventional gaming, exergaming significantly increased oxygen consumption (9.60 ± 0.50 vs. 5.05 ± 0.16 mL/kg/min) and energy expenditure (3.50 ± 0.14 vs. 1.92 ± 0.07 kcal/min), resulting in an almost doubling of caloric expenditure, without significant changes in heart rate or perceived exertion [46].

Another study compared running at self-selected intensity, running at moderate intensity (55% VO_2_ reserve), an active entertainment video game, and an active video game in 23 sedentary young adults (11 men and 12 women; mean age 24.8 ± 1 years). Oxygen consumption (VO_2_), heart rate, energy expenditure, METs, ventilatory threshold, perceived exertion, and psychological parameters such as flow state, pleasure, and psychological well-being were monitored. The fitness game induced heart rate values similar to conventional exercise (151.6 ± 2.1 bpm vs. 151.2 ± 3.6 bpm), with an energy expenditure of 8.5 ± 0.4 kcal/min and 6.4 ± 0.2 METs, while the entertainment game generated lower energy expenditure (6.7 ± 0.4 kcal/min; 5.1 ± 0.3 METs), but the highest enjoyment scores [47].

An interesting hypothesis that needs further detailed examination is whether competitive gaming which in some individuals (susceptible) will create a cycle of repeated psychological stress plus sympathetic nervous system (SNS)/HPA activation, might be sufficient to exacerbate significantly vascular/endothelial stress causing altered immune signaling and potentially chronic low-grade inflammation.

Sleep hygiene and circadian rhythm compliance can also influence cardiac performance. Thus, a study of 19 professional gamers (mean age 20.0 ± 1.6 years), monitored by Oura Ring and assessed by psychomotor vigilance tests (PVT), simple reaction time (SRT) and choice reaction time (CRT) in a character-based tactical shooter game and who were encouraged to increase their sleep duration by at least one hour per night for 4 weeks showed that although sleep duration did not increase significantly (408.8 vs. 418.5 min), they showed a significant increase in heart rate variability (HRV; *p* < 0.05), suggesting a possible improvement in cardiovascular autonomic regulation [14].

HRV is a marker of sympatho-vagal balance, and low values indicate predominance of sympathetic activity and reduced vagal tone, being associated with hypertension, arrhythmia and increased cardiovascular risk. Although there are no studies yet directly demonstrating endothelial dysfunction in esports players, acute increases in central arterial blood pressure and pulse wave velocity (PWV) during competitions suggest a transient increase in vascular tone and arterial stiffness. Repetition of these long-term responses may promote endothelial stress and increase cardiometabolic risk, particularly in individuals with preexisting risk factors.

Ketelhut and Nigg evaluated the cardiovascular and autonomic response induced by competitive gaming in 27 esports players (all males, 23.3 ± 2.8 years), 14 of whom played FIFA 21 and 13 played MOBA 5 vs. 5. The study measured HR, HRV; root mean square of successive differences (RMSSD) and standard deviation of normal-to-normal (SDNN), peripheral and central blood pressure, PWV, and energy expenditure (EE). During both gaming sessions, significant increases in HR, peripheral systolic blood pressure, diastolic blood pressure, central blood pressure, PWV), and energy expenditure were observed, concomitant with decreases in RMSSD, indicating reduced parasympathetic activity and predominance of sympathetic activation. No significant differences were observed between the two game genres, suggesting that cardiovascular stress is mainly determined by the competitive nature of gaming (and importantly, an individual’s response to competitive situations) and not by the type of game [48].

Another important aspect with a predictive role in cardiovascular pathology is the concept of slow cardiovascular recovery after stress. Thus, a study on the effects of stress induced by 30 min of challenge, threat and neutral gaming on the cardiovascular response in 135 young adults (49% men, 51% women; mean age 19 ± 4 years), showed that compared to neutral gaming, games with high vigilance requirements caused significant increases in blood pressure and heart rate, with the most pronounced changes being observed in the threat condition. In addition, participants in the threat group showed the slowest return of blood pressure to baseline values and cardiovascular activation was maintained throughout the 30 min of gaming, suggesting that sustained vigilance during gaming is associated with acute cardiovascular activation, providing a physiologically plausible link between gaming-related stress and cardiovascular vulnerability [15].

On the other hand, a study by Alesi et al. (2025) [49] showed that video games in the absence of intense competitive pressure do not necessarily induce predominantly sympathetic activation. Thus, in 31 young men (23.0 ± 3.5 years; gaming experience 8.7 ± 4.7 years; approximately 2 h of game play/day), subjected to a MOBA or FPS game for 120 min, on two consecutive days, compared to watching a nature documentary under identical experimental conditions, it was observed that HR and HRV parameters; RMSSD and HF-HRV did not change significantly and even more, watching the nature film significantly reduced heart rate and increased both RMSSD and HF-HRV, suggesting a parasympathetic activation and a superior relaxing effect to gaming [49].

HRV provides an indirect measure of cardiac autonomic regulation, while changes in central arterial blood pressure and pulse wave velocity during competitive gaming may reflect transient cardiovascular responses to psychological stress. Although these parameters have been associated with cardiovascular health in other populations, their acute alterations during gaming do not necessarily indicate persistent autonomic dysfunction, structural arterial stiffness, or increased long-term cardiovascular risk. Repeated exposure to these physiological responses, particularly when combined with prolonged sedentary behavior and preexisting cardiometabolic risk factors, may provide a plausible mechanism for cardiovascular vulnerability [50].

In the context of esports, prolonged sedentary behavior may compromise the physiological endocrine communication between skeletal muscle and adipose tissue, predisposing players to metabolic alterations associated with physical inactivity. Although no studies have directly demonstrated disruption of muscle-adipose tissue crosstalk in esports athletes, experimental models of prolonged sedentarism support the hypothesis that reduced muscle contraction progressively impairs myokine-adipokine signaling, favoring visceral adiposity, and the development of sarcopenic obesity, a recognized risk factor for future cardiovascular disease.

An experimental model of extreme sedentary behavior was developed by Dirks et al., who evaluated the effects of 7 days of complete bed rest in 10 healthy young men (23 ± 1 years). The study showed that after only 1 week of sedentary behavior, a loss of 1.4 ± 0.2 kg of lean mass, a 3.2 ± 0.9% reduction in quadriceps area, accompanied by a decrease in VO_2_peak of 6.4 ± 2.3%, muscle strength of 6.9 ± 1.4%, and insulin sensitivity of 29 ± 5% (all *p* < 0.05) were observed. These changes were associated with a reduction in muscle oxidative capacity, demonstrating that even short periods of physical inactivity rapidly induce muscle loss and metabolic alterations in young adults [22].

Lack of physical activity rapidly impairs skeletal muscle mitochondrial function. Gram et al. investigated 17 healthy young men (23.4 ± 0.5 years) and 15 elderly men (68.1 ± 1.1 years) who underwent a 2-week protocol of unilateral lower limb immobilization followed by 6 weeks of supervised aerobic training. Mitochondrial function was assessed from vastus lateralis muscle biopsies by measuring mitochondrial respiration, H_2_O_2_ production, membrane potential, and oxidative phosphorylation efficiency. Immobilization resulted in decreased ATP-generating respiration, concomitant with increased mitochondrial H_2_O_2_ emissions, indicating reduced oxidative efficiency and the onset of mitochondrial oxidative stress. These changes were largely reversible after the aerobic training period, suggesting that physical inactivity, rather than directly the age was the main determinant of mitochondrial dysfunction [23].

Confirming this phenomenon, a study that evaluated skeletal muscle mass, body fat percentage, and cardiorespiratory fitness in 147 healthy young adults (mean age 21 ± 2.16 years, 57% women) with a sedentary lifestyle (6–8 h/day spent sitting and <150 min of moderate-vigorous physical activity/week) showed that 41.5% of participants had presarcopenia, and 37.4% met the criteria for the sarcopenic obesity phenotype [24].

Although bed rest and limb immobilization represent more severe forms of muscle disuse than prolonged sitting during esports participation, these experimental models provide mechanistic evidence of the consequences of reduced skeletal muscle contractile activity, including impaired muscle protein homeostasis, mitochondrial function, and insulin sensitivity. Prolonged sedentary gaming may share some of these underlying mechanisms, particularly when accompanied by insufficient physical activity. This hypothesis is indirectly supported by observational studies reporting reduced daily physical activity and unfavorable body composition profiles in some esports populations. However, the magnitude of muscle disuse differs substantially between immobilization and sedentary gaming, and direct evidence linking esports participation to progressive muscle loss and sarcopenic obesity is currently lacking. Therefore, this proposed association should be regarded as mechanistically plausible but not experimentally established and requires validation through longitudinal studies. Figure 3 illustrates the molecular responses associated with physical activity and the potential alterations in metabolic, inflammatory, and tissue-specific signaling pathways under sedentary conditions.

## 6. Nitric Oxide Signaling and Endothelial Dysfunction in Chronic Gaming-Associated Stress

Nitric oxide (NO) signaling may also represent an important mechanistic link between prolonged sedentary behavior, chronic neuroendocrine stress, and cardiovascular vulnerability in esports players. NO is generated from L-arginine by nitric oxide synthase (NOS), of which endothelial NOS (eNOS) plays a central role in vascular homeostasis by promoting vasodilation, limiting platelet activation and leukocyte adhesion, and maintaining an anti-inflammatory and antithrombotic endothelial phenotype [51].

Prolonged sitting provides a particularly relevant mechanistic model for esports because reduced lower-limb movement decreases blood flow and endothelial shear stress. Low and oscillatory shear stress can increase endothelial reactive oxygen species (ROS) production while reducing eNOS expression and NO generation, whereas physiological laminar shear stress promotes eNOS-derived NO production and endothelial antioxidant signaling, effects that occur quite rapidly. Three hours of uninterrupted sitting can acutely impair vascular function by significantly reducing popliteal blood flow and impairing both endothelium-dependent flow-mediated dilation (FMD) and endothelium-independent nitroglycerin-mediated dilation (NMD) in young healthy adults. Nitrate-rich beetroot juice has been shown to increase plasma nitrate and NO metabolites and prevent the reduction in FMD [29].

In esports, the vascular effects of prolonged sitting may be further amplified by psychological and neuroendocrine stress. A total of 8 min of acute mental stress itself can transiently impair endothelial function by reducing brachial artery FMD 30 min after stress, with endothelial impairment persisting for up to 90 min under high-fat postprandial conditions [52].

These findings derive from experimental studies involving prolonged sitting or acute psychological stress in non-gaming populations. Although reductions in FMD indicate transient impairment of vascular reactivity, they do not establish persistent endothelial injury or atherosclerotic disease in esports participants. At the same time, the acute increases in blood pressure and PWV observed during competitive gaming reflect short-term hemodynamic responses rather than demonstrated structural vascular damage. Nevertheless, the potential cumulative effects of repeated exposure to psychological stress and prolonged sitting should be investigated in longitudinal studies assessing endothelial function and vascular health.

Oxidative stress can reduce NO bioavailability, while excessive oxidative stress may promote eNOS uncoupling, shifting the enzyme from NO toward superoxide production and thereby establishing a feed-forward cycle of oxidative stress and endothelial dysfunction. In 1986, the transactional theory of stress described stress as occurring when perceived demands exceed an individual’s ability to cope with them [53]. HPA-axis activation may also influence this pathway by reducing NO bioavailability through the suppression of eNOS expression and activity (Figure 4) [54].

Adipose tissue and skeletal muscle are endocrine organs that communicate bidirectionally through the secretion of adipokines, myokines, lipokines and cytokines with direct metabolic implications or by modifying insulin sensitivity, inflammatory status and mitochondrial function.

### 6.1. Adipokine and Myokine Dysregulation During Chronic Stress

Corticosteroids promote adipocyte differentiation and hypertrophy with visceral lipid accumulation, as demonstrated in a study conducted on 60-day-old male Sprague–Dawley rats that were chronically (2–7 days) administered low-dose dexamethasone. Corticosteroid administration directed adipocyte precursor cells (APCs) toward adipogenic differentiation, demonstrated by increased expression of adipogenic competence markers such as PPARγ2 and Zfp423, an effect that was abolished by simultaneous administration of GR and MR inhibitors [55]. Hypertrophic adipose tissue is metabolically dysfunctional and secretes increased levels of proinflammatory factors such as leptin, resistin, and MCP-1 and reduced levels of adiponectin, factors that together contribute overtime to insulin resistance [56].

Physical exercise positively influences adipokines levels as demonstrated in a clinical study of 62 overweight or obese children (30 boys and 32 girls) aged 6 to 9 years who were subjected to 16 weeks of moderate-intensity interval gaming (MIIG) or high-intensity interval gaming (HIIG) active game sessions. Salivary leptin levels decreased significantly after the intervention, TNF-α levels showed a trend towards reduction, while IL-6 and insulin levels suggested a possible insulin resistance in obese children [57]. On the other hand, a clinical study conducted on 14 male patients with pathological gambling, 11 patients with internet gaming disorder, and 39 patients with alcohol use disorder highlighted an inverse activating mechanism of leptin, correlated with BMI on the HPA axis in the context of compulsive behaviors and reward/stress mechanisms associated with gaming and gambling [58].

Unlike adipokines, myokines released during physical exercise have an anti-inflammatory role, favor browning of white adipose tissue, increase mitochondrial oxidative metabolism and improve glucose homeostasis. As evidence of the adaptability of adipose tissue and the attempt to compensate for muscle damage, a study conducted on 23 healthy volunteers of different ages in an experimental model of 14-day bed-rest showed that muscle mass and muscle performance decreased, circulating irisin increased significantly, while fibronectin type III domain-containing protein 5 (FNDC5) expression decreased in skeletal muscle but increased more than 2-fold in adipose tissue, thus adipose tissue becomes an important source of irisin and tries to compensate for muscle damage through a muscle-adipose tissue crosstalk mechanism [59].

### 6.2. Disruption of Muscle–Adipose Tissue Crosstalk as a Driver of Sarcopenic Obesity

Muscle and adipose tissue form an interconnected endocrine network, which during exercise leads to the release of myokines such as irisin, muscle IL-6 or IL-15 that favor oxidative metabolism and browning of adipose tissue. At the same time, adipose tissue secretes adipokines such as leptin and adiponectin that regulate appetite, insulin sensitivity, inflammation and energy metabolism. Under resting conditions (bed rest) with complete isolation for 14 days, 23 healthy male volunteers, including 7 young people (23.3 ± 2.8 years) and 16 older adults (59.3 ± 3.0 years), showed increased fat mass (+1.4 kg), decreased fat-free mass (−4.1 kg), reduced quadriceps volume (−133.9 cm^3^), decreased maximum voluntary muscle force (−69 N) and a significant increase in plasma irisin concentration, while FNDC5 expression decreased in muscle but increased 2.3-fold in adipose tissue suggesting that, under conditions of prolonged inactivity, adipose tissue becomes a compensatory source of irisin, the basis of this crosstalk [59].

Therefore, although experimental evidence supports the involvement of the sedentary–stress–eNOS/NO axis in the pathophysiology of cardiovascular disease in adults, long-time prospective studies are needed to determine whether alterations in this pathway represent a potential cardiovascular risk factor in young individuals engaged in esports, either recreationally or professionally.

## 7. Inflammation, Endothelial Dysfunction, and Cardiovascular Risk in Chronic Gaming-Associated Stress

Prolonged sedentary behavior and recurrent neuroendocrine stress, two characteristic features of esports, may converge to promote chronic low-grade inflammation, a key pathophysiological process involved in the development and progression of cardiovascular disease [60].

The first factor implicated in the development of a pro-inflammatory phenotype is the continuous activation of the sympathetic nervous system during periods of stress. A single 3 h episode of psychological stress induced by restraint in mice rapidly and transiently decreased the number of circulating leukocytes, particularly inflammatory monocytes and lymphocytes. Myeloid cells fluorescently labeled with GFP and reinjected into mice were redistributed from the circulation to the skin, heart, and lungs. At the same time, excised aortas from wild-type mice subjected to 30 min of mental stress showed rapid endothelial cell activation, with increased intercellular adhesion molecule (ICAM1) and vascular cell adhesion molecule (VCAM1) protein levels [61].

Primary human monocytes exposed in vitro to norepinephrine or epinephrine for 24 h and subsequently differentiated into macrophages exhibited increased glycolysis and oxidative phosphorylation. Upon subsequent stimulation with LPS, the same cells produced significantly more TNF-α [62].

Lack of physical activity is the second factor contributing to vascular damage. A total of 180 min of uninterrupted sitting or sitting interrupted by 10 calf raises every 10 min resulted in decreased circulating microparticle populations associated with circulating angiogenic cells (CAC) paracrine activity, endothelial activation, and apoptosis (annexin V^+^/CD34^+^, annexin V^+^/CD62E^+^, and annexin V^+^/CD31^+^/CD42b^−^, respectively), whereas circulating CD14^+^/CD31^+^, CD3+/CD31^+^, and CD34^+^ angiogenic cell populations associated with endothelial repair, as well as plasma endothelin-1 concentrations, remained unchanged [63]. Although these findings indicate that prolonged sitting may alter circulating microparticle profiles, such changes should not be interpreted as direct evidence of endothelial injury or vascular dysfunction. Nevertheless, they may reflect early alterations in endothelial homeostasis, the physiological significance of which requires further investigation.

Prolonged sitting acutely impairs both endothelium-dependent and NMD. After 3 h of prolonged sitting, healthy adults (22 ± 2 years) exhibited significant reductions in popliteal blood flow, FMD, and NM [29].

The interaction between psychological stress and physical inactivity may represent a biologically plausible mechanism contributing to cardiovascular vulnerability, although its direct association with long-term cardiovascular risk in esports participants remains to be established. Regular physical activity may reduce the inflammatory and vascular response to acute psychological stress. Atherosclerosis-prone mice, subjected to physical activity presented attenuated stress-induced leukocyte redistribution and reduced recruitment of inflammatory myeloid cells into atherosclerotic plaques. Active mice also exhibited lower endothelial ICAM1 and ICAM2 protein levels and reduced vascular expression of *Vcam1*, *Icam1*, *Ccl2*, *Ccl3*, and *Ccl5* following stress. Circulating leukocytes and inflammatory CD14+/CD16− monocytes decreased in physically inactive healthy humans exposed to acute emotional stress, but remained unchanged in physically active individuals [30].

Although long-term experimental studies assessing inflammatory biochemical markers in young gamers are currently lacking, several mechanistic parallels can be drawn from existing studies in adults, particularly those exposed to prolonged sedentary behavior and psychologically demanding desk-based work.

Future prospective studies should therefore extend beyond the assessment of short-term responses to gaming in young individuals and investigate the long-term consequences of recurrent neuroendocrine stress and prolonged sitting, particularly in individuals who engage in prolonged gaming outside working hours, either alone or in combination with sedentary desk-based occupations. Longitudinal studies are needed to determine whether this cumulative exposure contributes to persistent vascular and metabolic alterations and ultimately increases the risk of chronic diseases, particularly cardiovascular disease, later in life.

## 8. Esports-Specific Features and Testable Predictions of the Sedentary–Stress Framework

The neuroendocrine, metabolic, and vascular consequences of sedentary behavior and psychological stress are recognized. Competitive esports may represent a distinct context in which these exposures converge.

This framework generates three experimentally testable predictions:

**(i) Competitive stress and neuroendocrine activation.** Competitive gaming is hypothesized to induce greater sympathetic and HPA-axis activation than noncompetitive gaming or duration-matched sedentary cognitive tasks. This could be tested using randomized crossover protocols assessing salivary cortisol, heart rate, heart rate variability (particularly RMSSD), and blood pressure before, during, and after gaming sessions.

**(ii) Movement breaks and vascular–metabolic responses.** Prolonged gaming without movement breaks may induce less favorable vascular and metabolic responses than identical gaming sessions incorporating brief periods of physical activity. Controlled crossover studies could compare continuous gaming with gaming interrupted by 3–5 min walking breaks every 30–60 min, measuring popliteal blood flow, flow-mediated dilation, blood pressure, glucose, and insulin responses.

**(iii) Chronic exposure and cardiometabolic adaptations.** Repeated competitive stress combined with prolonged sedentary behavior and insufficient sleep may be associated with adverse changes in metabolic regulation, body composition, and endothelial function. Prospective studies following esports athletes and appropriately matched sedentary controls over 6–12 months could assess objectively measured physical activity and sleep, repeated cortisol and HRV measurements, insulin resistance (HOMA-IR), lipid profiles, body composition (DXA), muscle strength, and flow-mediated dilation. Such studies would help determine whether the proposed sedentary–stress phenotype is associated with measurable cardiometabolic alterations over time.

The physiological consequences of competitive gaming may be influenced by several individual and lifestyle-related factors that should be considered when interpreting esports-related cardiometabolic outcomes. Physical activity performed outside gaming sessions may counteract some of the adverse effects associated with prolonged sitting, whereas dietary patterns, caffeine consumption, and energy drink intake may independently influence glucose metabolism, sympathetic activation, heart rate, and blood pressure. Chronotype and habitual sleep schedules may further modify neuroendocrine and circadian responses, particularly among players engaging in late-night gaming. In addition, body mass index, pre-existing metabolic or cardiovascular conditions, and other comorbidities may influence baseline cardiometabolic profiles and individual susceptibility to physiological stress. These variables should be systematically assessed and accounted for in future esports studies to distinguish gaming-associated effects from lifestyle-related and clinical confounding factors.

## 9. Limitations

This narrative review has several limitations. First, its non-systematic design does not allow for a comprehensive quantitative assessment of the available evidence and may introduce selection bias. Second, the proposed relationships between prolonged gaming, neuroendocrine stress, metabolic dysregulation, and endothelial dysfunction are primarily based on the integration of findings from different experimental and observational settings rather than direct mechanistic investigations in esports athletes. Third, the limited availability of randomized controlled trials, prospective longitudinal studies, and standardized gaming exposure protocols restricts the establishment of causal relationships. Furthermore, considerable heterogeneity in gaming duration, competitive intensity, participant characteristics, and physiological assessment methods limits comparisons across studies. Finally, the potential long-term cardiovascular consequences of the proposed “stress without movement” phenotype remain hypothetical and require validation through prospective studies incorporating neuroendocrine, metabolic, and vascular biomarkers.

## 10. Conclusions

Prolonged gaming may represent a “stress without movement” phenotype in which recurrent neuroendocrine activation combined with physical inactivity promotes metabolic and endothelial vulnerability, potentially increasing the long-term risk of sarcopenic obesity and cardiovascular disease. Reducing uninterrupted sitting, incorporating regular physical activity, and maintaining adequate sleep and circadian hygiene should represent the primary preventive strategies, while pharmacological prevention is not currently supported in otherwise healthy young individuals.

An important unresolved question is whether the physiological consequences of gaming differ according to the balance between physical exertion and psychological stress. Conventional exercise such as running induces substantial metabolic and cardiovascular stress accompanied by muscle contraction, increased oxygen consumption and transient inflammatory signaling that can contribute to subsequent physiological adaptation. In contrast, competitive gaming can elicit marked psychological, sympathetic and, in some settings, neuroendocrine activation despite minimal physical activity.

Physically active or “exergaming” environments may represent a further distinct phenotype, combining exercise-related metabolic stress with the cognitive and competitive stress of gaming. Although there is currently insufficient evidence to conclude that this combined response is more inflammatory or detrimental than conventional exercise, the simultaneous activation of physical and psychological stress pathways could produce a different neuroendocrine, vascular and immune response. Determining whether competitive active gaming generates a qualitatively or quantitatively different inflammatory and endothelial response from conventional exercise therefore represents an important area for future investigation.

## Figures and Tables

**Figure 1:**
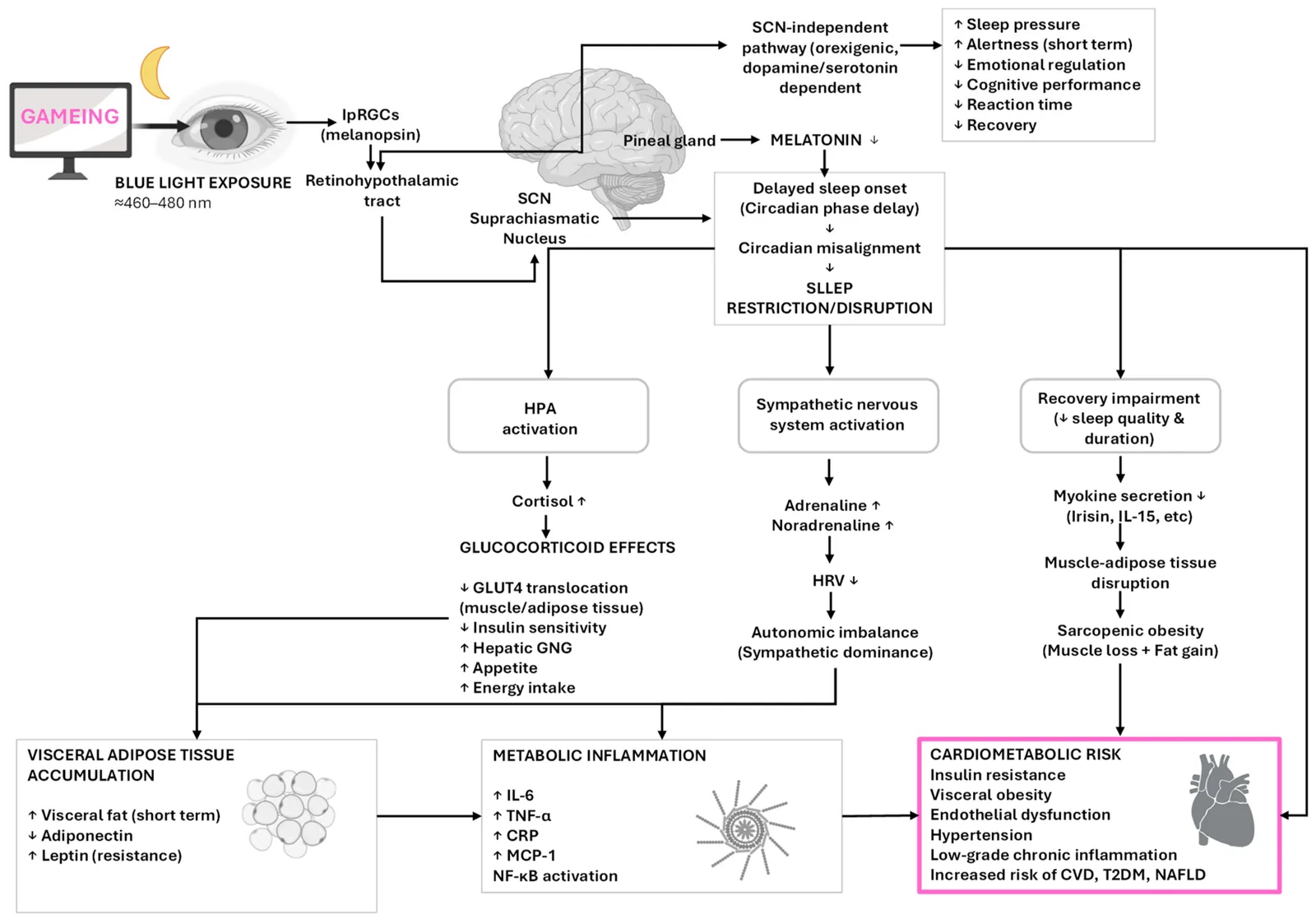
Proposed Neuroendocrine and Metabolic Pathways Linking Prolonged Gaming to Cardiometabolic Vulnerability. Abbreviations: IL-6—interleukin-6, IL-15—interleukin-15, TNF-α—tumor necrosis factor-alpha, CRP—C-reactive protein, MCP-1—monocyte chemoattractant protein-1, NF-κB—nuclear factor kappa B, CVD—cardiovascular disease, T2DM—type 2 diabetes mellitus, NAFLD—non-alcoholic fatty liver disease, GLUT4—glucose transporter type 4, GNG—gluconeogenesis, HRV—heart rate variability, HPA—hypothalamic–pituitary–adrenal axis, ipRGCs—intrinsically photosensitive retinal ganglion cells, SCN—suprachiasmatic nucleus, ↑ up arrow means increased effect, ↓ down arrow means decreased effect. Created in BioRender. Tero-Vescan, A. (2026) https://BioRender.com/ziuc7n1.

**Figure 2:**
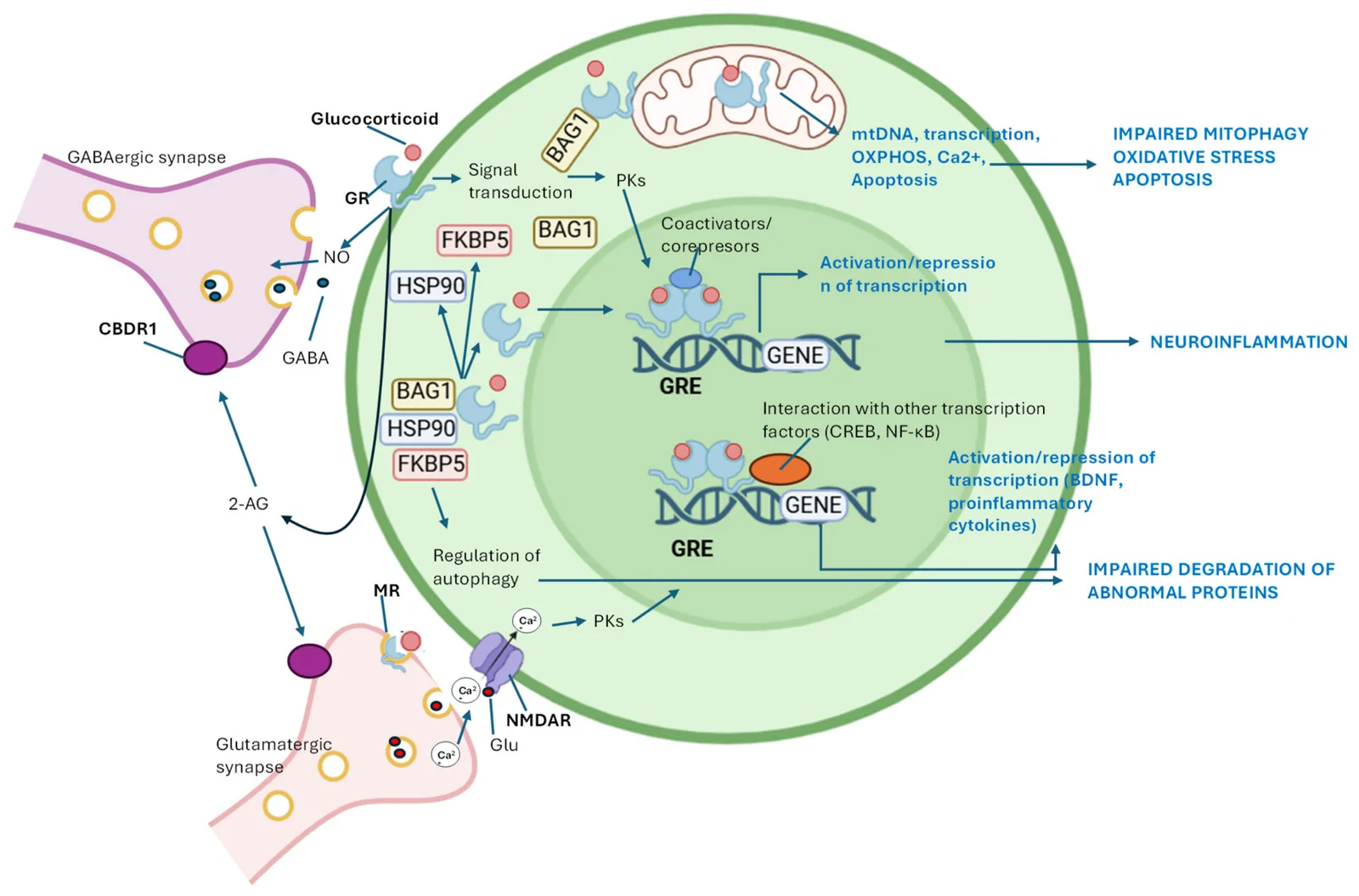
Neurobiological and molecular mechanisms of chronic glucocorticoid exposure. Abbreviations: GR—glucocorticoid receptor, MR—mineralocorticoid receptor, BAG1—BCL2-associated athanogene 1, BDNF—brain-derived neurotrophic factor, Ca^2+^—calcium ion, CREB—cAMP response element-binding protein, FKBP5—FK506-binding protein 5, GABA—gamma-aminobutyric acid, GABAR—gamma-aminobutyric acid receptor, Glu—glutamate, GRE—glucocorticoid response element, HSP90—heat shock protein 90, mtDNA—mitochondrial DNA, NF-κB—nuclear factor kappa B, NMDAR—N-methyl-D-aspartate receptor, NO—nitric oxide, OXPHOS—oxidative phosphorylation, PKs—protein kinases. Created in BioRender. Tero-Vescan, A. (2026) https://BioRender.com/p8bg8ep.

**Figure 3:**
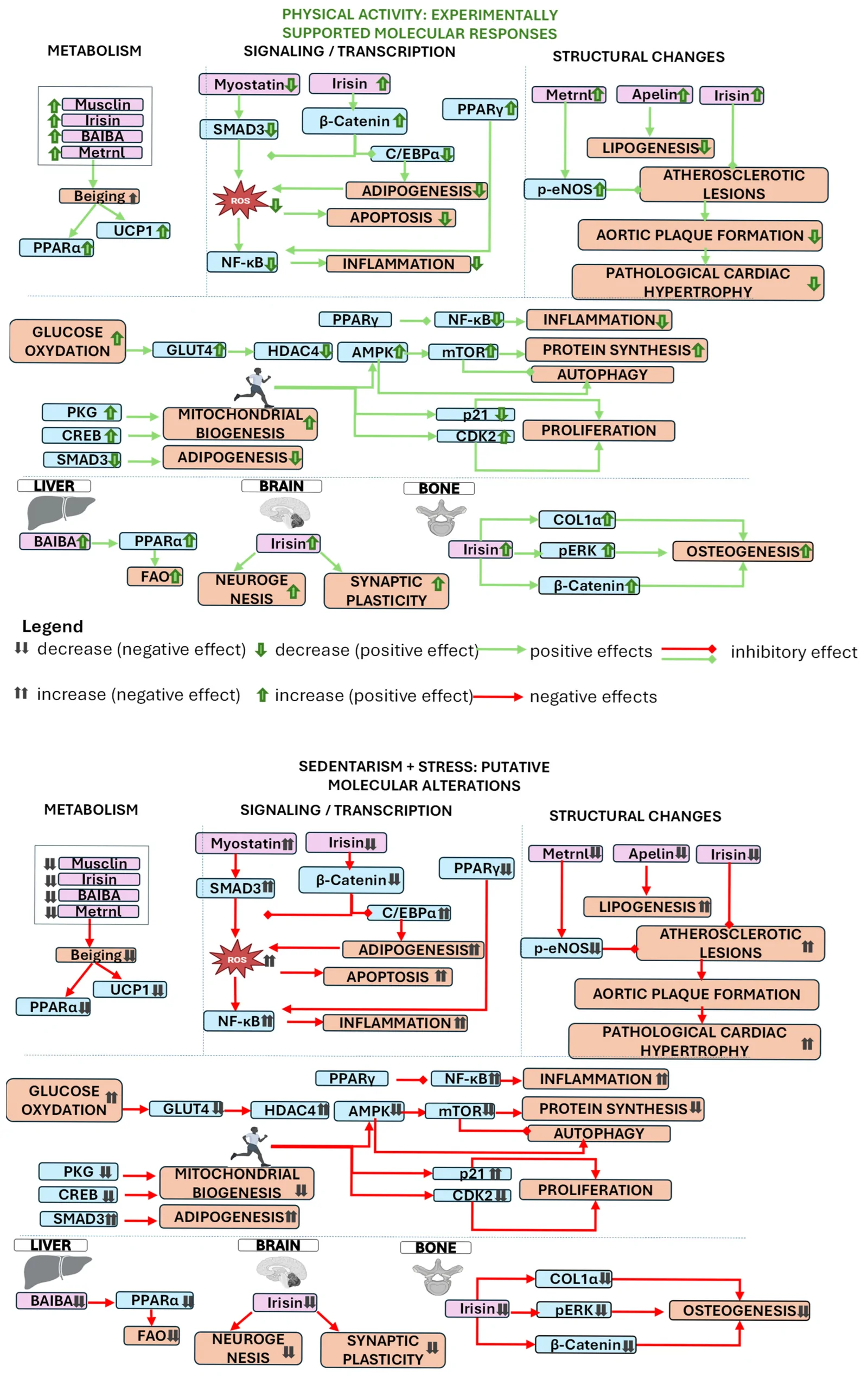
Established exercise-related molecular responses and putative alterations associated with combined sedentary behavior and psychological stress. Abbreviations BAIBA—β-aminoisobutyric acid, C/EBPα—CCAAT/enhancer-binding protein alpha, CDK2—cyclin-dependent kinase 2, COL1α—collagen type I alpha, CREB—cAMP response element-binding protein, FABP4—fatty acid-binding protein 4, FAO—fatty acid oxidation, GLUT4—glucose transporter type 4, HDAC4—histone deacetylase 4, IGF-1—insulin-like growth factor 1, Metrnl—meteorin-like protein, mTOR—mechanistic target of rapamycin, NF-κB—nuclear factor kappa B, p-eNOS—phosphorylated endothelial nitric oxide synthase, pERK—phosphorylated extracellular signal-regulated kinase, PKG—protein kinase G, PPARα—peroxisome proliferator-activated receptor alpha, PPARγ—peroxisome proliferator-activated receptor gamma, ROS—reactive oxygen species, SMAD3—SMAD family member 3, UCP1—uncoupling protein 1.

**Figure 4:**
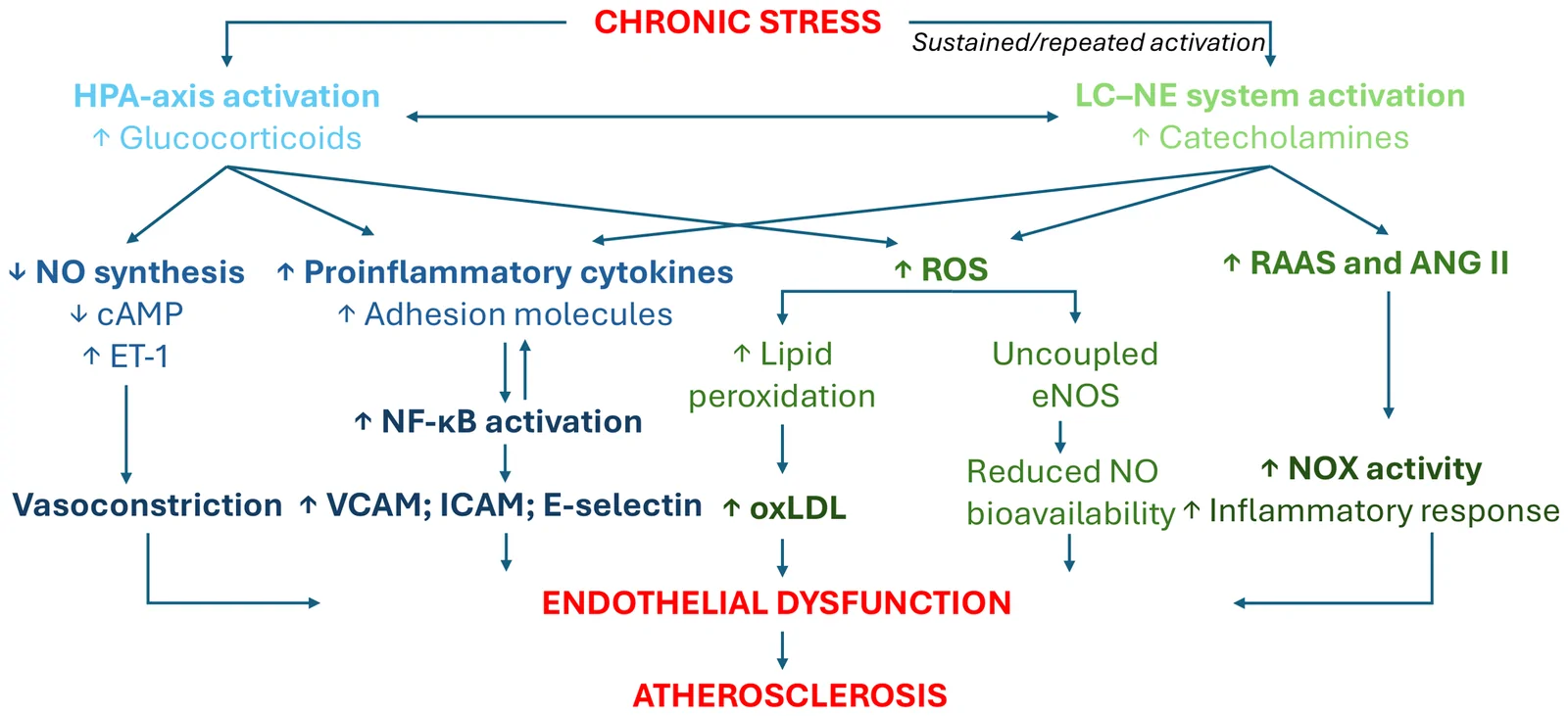
Molecular mechanisms link chronic stress to endothelial dysfunction and atherosclerosis. Abbreviations: ANG II—angiotensin II, cAMP—cyclic adenosine monophosphate, eNOS—endothelial nitric oxide synthase, ET-1—endothelin-1, HPA—hypothalamic–pituitary–adrenal, ICAM—intercellular adhesion molecule, LC–NE—locus coeruleus–norepinephrine, NF-κB—nuclear factor kappa B, NO—nitric oxide, NOX—NADPH oxidase, oxLDL—oxidized low-density lipoprotein, RAAS—renin–angiotensin–aldosterone system, ROS—reactive oxygen species, VCAM—vascular cell adhesion molecule, ↑ up arrow means increased effect, ↓ down arrow means decreased effect.

**Table 1:** Hierarchy of evidence supporting the proposed neuroendocrine, metabolic, and endothelial mechanisms in esports.

Mechanism	Direct Esports/Gaming Evidence	Indirect Human or Experimental Evidence	Interpretation	Ref.
HPA axis activation	Acute salivary cortisol responses during gaming competitions	Experimental glucocorticoid exposure and stress models	Acute HPA responses documented; chronic consequences unconfirmed	[9,10,11,12,13]
Autonomic and cardiovascular activation	HR, HRV, BP, and PWV responses during gaming	Experimental psychological stress	Acute cardiovascular responses documented; long-term effects uncertain	[14,15]
Sleep and circadian disruption	Sleep duration, quality, and timing assessed in gamers	Experimental sleep restriction in non-gaming populations	Gaming-related sleep alterations documented; downstream metabolic consequences partly extrapolated	[16,17,18,19,20]
Skeletal muscle inactivity and sarcopenic obesity	Physical activity and body composition observations in esports participants	Bed rest, limb immobilization, and sedentary population studies	Reduced activity documented; progressive muscle loss/sarcopenic obesity not established	[21,22,23,24]
Glucose and adipose metabolic dysfunction	Limited direct measurements of metabolic responses during gaming	Glucocorticoid-treated adipocytes and animal models	Molecular pathways supported experimentally, but not directly validated in esports	[12,13,25,26,27,28]
Endothelial dysfunction	Acute hemodynamic responses; limited direct endothelial measurements	Prolonged sitting and experimental psychological stress	Potential vascular mechanism; chronic endothelial dysfunction in esports unconfirmed	[15,29,30]
Long-term cardiovascular outcomes	No prospective esports-specific cardiovascular event evidence identified	Cardiovascular studies in non-gaming populations	Hypothetical association, not clinically established	[6,15,29]

**Table 2:** Representative experimental studies comparing psychophysiological stress responses between esports athletes, recreational gamers, and control participants.

Study Type	Participants/Age	Game/Competitive Context	Duration/Exposure	Cortisol (Saliva) Pregame	Cortisol (Saliva) Postgame	Comments	Ref.
Amateur players	19 male and 4 female 23.7 ± 3.3 ys	MOBA/Tactical shooter game	35 ± 22 min	3.2 ± 1.9 nmol/L winners 3.7 ± 2.4 nmol/L losers	winners 8.2 ± 5.5 nmol/L losers 7.1 ± 7.2 nmol/L	inverted U-shaped relationship between physiological arousal and anxiety with game performance	[9]
	30 participants 23.1 ± 3.0 years	FPS multiplayer/soccer simulation game	30 min	3.1 ± 2.9 ng/mL	2.2 ± 2.3 ng/mL	no significant changes in oxygen uptake, carbon dioxide output, energy expenditure, stroke volume, or lactate levels for 30 min intervention	[12]
Esports athletes vs. nonplayers	25 expert esports players and 20 controls 18–27 years	MOBA	30–60 min	Esports athletes 3.75 ± 1.76 ng/mL Controls 2.68 ± 1.48 ng/mL	Esports athletes 3.83 ± 2.30 ng/mL Controls 2.85 ± 1.35 ng/mL	pronounced anticipatory psychophysiological stress response before competition in esports athletes	[11]

Abbreviations: MOBA—multiplayer Online Battle Arena, FPS—first person shooter.

## Data Availability

No new data were created or analyzed in this study. Data sharing is not applicable to this article.

## Abbreviations

The following abbreviations are used in this manuscript:

11β-HSD1	11β-hydroxysteroid dehydrogenase type 1
Akt	protein kinase B
AMPK	AMP-activated protein kinase
ANG II	angiotensin II
APCs	adipocyte precursor cells
BAG1	BCL2-associated athanogene 1
BAIBA	β-aminoisobutyric acid
BDNF	brain-derived neurotrophic factor
BMI	body mass index
C/EBPα	CCAAT/enhancer-binding protein alpha
CAC	circulating angiogenic cells
cAMP	cyclic adenosine monophosphate
CDK2	cyclin-dependent kinase 2
COL1α	collagen type I alpha
CREB	cAMP response element-binding protein
CRP	C-reactive protein
CRT	choice reaction time
CVD	cardiovascular disease
ECP	exercise cardiac power
eNOS	endothelial nitric oxide synthase
ET-1	endothelin-1
FABP4	fatty acid-binding protein 4
FAO	fatty acid oxidation
FKBP5	FK506-binding protein 5
FMD	flow-mediated dilation
FNDC5	fibronectin type III domain-containing protein 5
FPS	first-person shooter
GABA	gamma-aminobutyric acid
GABAR	gamma-aminobutyric acid receptor
Glu	Glutamate
GLUT4	glucose transporter type 4
GNG	gluconeogenesis
GR	glucocorticoid receptor
GREs	glucocorticoid response elements
GRR	glucocorticoid receptor resistance
HDAC4	histone deacetylase 4
HIIG	high-intensity interval gaming
HPA	hypothalamic–pituitary–adrenal
HPG	hypothalamic-pituitary-gonadal
HRV	heart rate variability
HSL	hormone-sensitive lipase
HSP90	heat shock protein 90
ICAM	intercellular adhesion molecule
IGF-I	insulin-like growth factor I
IL-6	interleukin-6
ipRGCs	intrinsically photosensitive retinal ganglion cells
IRS-1	insulin receptor substrate-1
MCP-1	monocyte chemoattractant protein-1
Metrnl	meteorin-like protein
MIIG	moderate-intensity interval gaming
MOBA	multiplayer online battle arena
MR	mineralocorticoid receptor
mtDNA	mitochondrial DNA
mTOR	mechanistic target of rapamycin
NAFLD	non-alcoholic fatty liver disease
NF-κB	nuclear factor kappa B
NMD	nitroglycerin-mediated dilation
NMDAR	N-methyl-D-aspartate receptor
NO	nitric oxide
NOS	nitric oxide synthase
NOX	NADPH oxidase
oxLDL	oxidized low-density lipoprotein
OXPHOS	oxidative phosphorylation
p-eNOS	phosphorylated endothelial nitric oxide synthase
PEPCK	phosphoenolpyruvate carboxykinase
pERK	phosphorylated extracellular signal-regulated kinase
PI3K	phosphoinositide 3-kinase
PKG	protein kinase G
PKs	protein kinases
PPARα	peroxisome proliferator-activated receptor alpha
PPARγ	peroxisome proliferator-activated receptor gamma
PSQI	Pittsburgh sleep quality index
PTSD	post-traumatic stress disorder
PVT	psychomotor vigilance tests
PWV	pulse wave velocity
RAAS	renin–angiotensin–aldosterone system
ROS	reactive oxygen species
RPG	role-playing game
SCN	suprachiasmatic nucleus
SMAD3	SMAD family member 3
SNS	sympathetic nervous system
SRT	simple reaction time
T2DM	type 2 diabetes mellitus
TNF-α	tumor necrosis factor-alpha
UCP1	uncoupling protein 1
VCAM	vascular cell adhesion molecule
VO_2_max	maximal oxygen uptake
WASO	wake after sleep onset
